# Ferritin polarization and iron transport across monolayer epithelial barriers in mammals

**DOI:** 10.3389/fphar.2014.00194

**Published:** 2014-08-25

**Authors:** Esther G. Meyron-Holtz, Lyora A. Cohen, Lulu Fahoum, Yael Haimovich, Lena Lifshitz, Inbar Magid-Gold, Tanja Stuemler, Marianna Truman-Rosentsvit

**Affiliations:** Laboratory for Molecular Nutrition, Faculty of Biotechnology and Food Engineering, Technion – Israel Institute of Technology, Technion CityHaifa, Israel

**Keywords:** iron transport, iron metabolism, epithelial barriers, tight junctions, ferritin polarization

## Abstract

Epithelial barriers are found in many tissues such as the intestine, kidney and brain where they separate the external environment from the body or a specific compartment from its periphery. Due to the tight junctions that connect epithelial barrier-cells (EBCs), the transport of compounds takes place nearly exclusively across the apical or basolateral membrane, the cell-body and the opposite membrane of the polarized EBC, and is regulated on numerous levels including barrier-specific adapted trafficking-machineries. Iron is an essential element but toxic at excess. Therefore, all iron-requiring organisms tightly regulate iron concentrations on systemic and cellular levels. In contrast to most cell types that control just their own iron homeostasis, EBCs also regulate homeostasis of the compartment they enclose or the body as a whole. Iron is transported across EBCs by specialized transporters such as the transferrin receptor and ferroportin. Recently, the iron storage protein ferritin was also attributed a role in the regulation of systemic iron homeostasis and we gathered evidence from the literature and original data that ferritin is polarized in EBC, suggesting also a role for ferritin in iron trafficking across EBCs.

## SELECTED PROTEINS INVOLVED IN IRON TRAFFICKING

In the blood stream, iron normally circulates bound to transferrin and is taken up by cells through binding of diferric transferrin to the transferrin receptor 1 (TfR1). This complex is internalized via clathrin-coated pits that form early endosomes. These are acidified, iron is released from transferrin, apo-transferrin is recycled to the plasma membrane (PM) and is released to the blood-stream (Trowbridge et al., 1993). The endosomally released iron undergoes reduction by the endosomal ferric-reductase Steap3 and is transported from the endosome to the cytosol by the divalent metal transporter 1 (DMT1), an H^+^/iron cotransporter (Picard et al., 2000; von Drygalski and Adamson, 2013). DMT1 is also found on the PM and in other subcellular locations where it can import iron into the cytosol. Imported iron may enter: (1) the cytosolic labile iron-pool, (2) different cellular compartments where it will be integrated to functional heme-, iron-sulfur cluster- or other iron-containing proteins, (3) ferritin, the intracellular iron storage protein. Imported iron may also be released from the cell via ferroportin.

Due to the potential toxicity of iron, uptake, storage, and mobilization pathways are tightly regulated. iron regulatory proteins (IRP) 1 and 2 regulate iron uptake and storage by binding to mRNA structures called iron regulatory elements (IRE). Iron release from cells is regulated by the iron-regulated hormone hepcidin, which controls ferroportin levels. Serum hepcidin concentration is regulated by many signals including iron, oxygenation and inflammation (Napier et al., 2005; Richardson et al., 2010).

## EPITHELIAL BARRIERS

To cross a monolayer of an epithelial barrier, a molecule or element must: (1) Reach the EBC, (2) Enter it, (3) Get across, and (4) Be exported on the other side. EBCs are connected with tight junctions that separate between the apical and basolateral membrane of the EBC and thus EBCs create a living cellular barrier. The apical and basolateral poles face completely different environments. EBCs are able to sense the two environments and transport nutrients and other molecules across according to the received signals. This depends on close interaction and crosstalk between EBCs and their neighboring cells. The direction of transport is dictated in part by the expression of transporters and receptors on the respective membrane. Most of these carriers have a default membrane to which they are trafficked, but in specific epithelial barriers the trafficking machinery has adapted to enable a change of direction. TfR1 has been used extensively as a marker for recycling-endosome trafficking and information on its localization is available in various cell-types.

## IRON TRAFFICKING PROTEINS IN SELECTED EPITHELIAL MONOLAYERS

**FIGURE 1:**
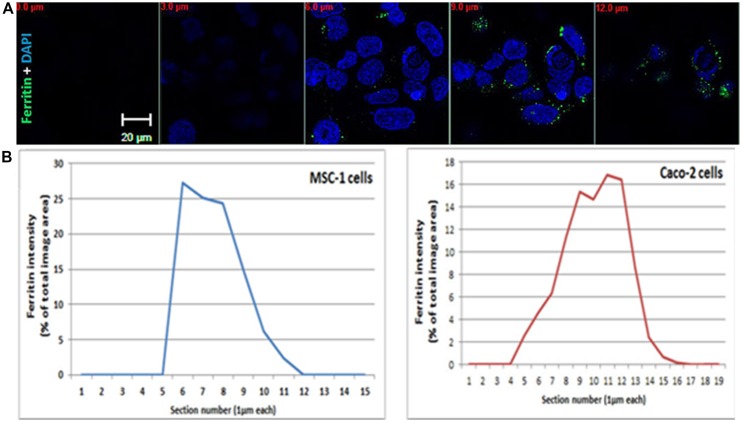
**Ferritin polarization. (A)** Confocal microscopy images of Caco2 cells stained with anti L-subunit ferritin antibodies (green) and nuclei are stained with DAPI (blue). Ferritin levels were higher between the 8–12 μm sections showing the apical side of the cells. Optical section-size is indicated as distance from glass-bottom in red. Laser power, voltage and offset were identical between different sections for each fluorophore. **(B)** Quantification of ferritin-fluorescence in MSC-1 and Caco2 cells is expressed as percent of total fluorescence of the image area. Each confocal slice is numbered. Number 1 indicates the coverslip side. The percent area occupied by the ferritin signal in each Z slice was calculated from the sum of fluorescence in all stacks and plotted as a function of confocal slice. An increase of intensity is clearly detected on the basolateral side of the MSC-1 and the apical side of the Caco-2 cells.

**FIGURE 2:**
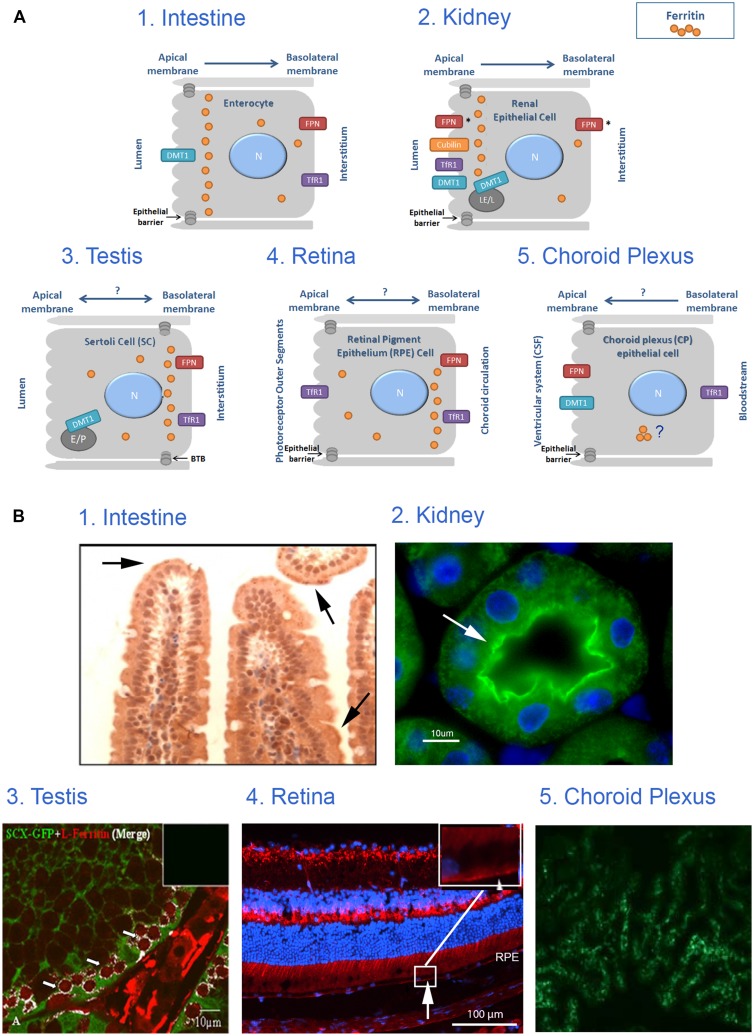
**Iron transport across monolayer epithelial barriers and subcellular ferritin polarization. (A)** Illustration of ferritin cellular polarization and iron reabsorption and trafficking across the epithelial barriers found in intestinal, renal, testicular, retinal and choroid plexus (CP) cells. Iron transporters are localized on the relevant membrane (N, nucleus; E, endosome; LE, late endosome; L, lysosome; P, phagosome; FPN, ferroportin; *represents suggested ferroportin localizations by the literature, arrows represent proposed direction of iron transport). **(B)** Intracellular ferritin polarization in epithelial barriers published in the literature. (1) In testinal villi paraffin sections stained with rabbit anti-ferritin H antibody (from Vanoaica et al., 2010) and reveal that ferritin is not evenly distributed throughout the cytosol, but appears in a punctate pattern that is more concentrated near the apical pole (arrows). (2) Kidney cortex sections stained with rabbit anti-ferritin H antibody (green). Nuclei were stained with DAPI (blue), (from Cohen et al., 2010) and reveals an uneven ferritin distribution in renal proximal tubule cells where ferritin was co-localized with villin (arrow) and was enriched near the apical pole of the PCT-cells. (3) Testis sections stained with rabbit anti-ferritin L antibody (red) and polyclonal GFP antibody (green), (from Leichtmann-Bardoogo et al., 2012), and reveals that ferritin is enriched in the SC cytosol close to the basolateral pole (white), especially around the early primary spermatocyte. (4) Retinal samples stained with rabbit anti-ferritin L antibody (red). Nuclei were stained with DAPI (blue; unpublished data, Joshua Dunaief, personal communication). This image reveals that ferritin is distributed in a polarized manner and was localized to the basal RPE (arrow) in a WT C57BL6/J retina. (5) Choroid plexus samples stained with anti-ferritin antibody (green), (from Rouault et al., 2009) shows expression of ferritin in 24-months old mouse-choroid plexus, but subcellular location cannot be determined. Permissions have been obtained for use of copyrighted material from all these sources.

Examining the location of TfR1, DMT1 and ferroportin in different epithelial monolayers revealed that each of these barriers has adapted iron trafficking to its environment and specific function. Interestingly, we found evidence that ferritin distribution is also affected by cell polarization (Figures 1 and 2B). Accumulation of ferritin at one end of a polarized cell can be due to trafficking of ferritin itself or a subcellular compartment in which ferritin is contained. In addition, ferritin may be taken up and endocytosed by one of the recently described ferritin receptors and endosomal ferritin may not be distributed evenly throughout the cell.

## INTESTINE

The duodenum is responsible for digestion and absorption of most nutrients including iron, while the jejunum and ileum mainly absorb nutrients that were not absorbed earlier. Dietary iron reaches the duodenal enterocytes either as ferric- or heme-iron. Ferric iron is reduced to ferrous iron by dietary components, such as amino acids, amines and ascorbic acid, or by ferric reductases of the brush border prior to absorption. Ferric iron can also be absorbed after chelation by mucins, which maintain the iron in the ferric state. Around 25 to 50% of dietary heme-iron is absorbed compared to only 1 to 10% of ionic iron and the two forms do not compete (Roy and Enns, 2000; Shayeghi et al., 2005; Ma et al., 2006; Sharp and Srai, 2007; MacKenzie et al., 2008; Le Blanc et al., 2012).

Ferrous- and heme-iron enter the enterocyte apically via DMT1 and possibly the heme-carrier-protein1 (HCP1), respectively (Gunshin et al., 1997; Le Blanc et al., 2012). Following absorption, heme is detectable in membrane bound vesicles within the cytoplasm. Heme-oxygenase1 (HO1) removes the iron from the protoporphyrin ring and the ferrous iron joins the intracellular pool along with non-heme-iron. The mechanism by which iron is translocated within the enterocyte, i.e., from the apical to the basolateral membrane, has not yet been elucidated.

During translocation, iron maintains its solubility and low reactivity possibly by binding to protein chaperones or to ferritin (Ma et al., 2006). Eﬄux of iron across the basolateral membrane is mediated by ferroportin, which is regulated systemically by hepcidin and locally by ferritin (Vanoaica et al., 2010). Following export, iron is oxidized by hephaestin and loaded onto transferrin for transport in the blood circulation (MacKenzie et al., 2008). If systemic signaling causes downregulation of ferroportin, enterocytes will fill up with ferritin and eventually slough into the lumen (Sharp and Srai, 2007). Interestingly, ferritin distribution in enterocytes is not even throughout the cytosol, but appears in a punctate pattern that is more concentrated near the apical pole (Figure 2B from Vanoaica et al., 2010) and Figure 1. TfR1 is expressed basolaterally in enterocytes, securing a continuous iron supply for these fast-dividing cells.

## KIDNEY

One of the many functions of the kidney is the protein- and nutrient-reabsorption from the primary urine. This takes place in the nephron, the basic unit of the kidney, which is composed of four main areas: glomerular capsule, proximal convoluted tubule (PCT), loop of Henle, and the distal convoluted tubule (DCT). Most reabsorption occurs in the PCT where epithelial cells apically express microvilli. In patients suffering from proteinuria, transferrin is abundant in the urine, suggesting that transferrin passes the glomerulus into the primary filtrate (Kozyraki et al., 2001). As the final urine contains neither protein nor iron, there must be a mechanism by which both transferrin and the bound iron are reabsorbed. In most tissues, transferrin-iron uptake is mediated by TfR1 and after intracellular iron release, apo-transferrin is recycled and released extracellularly (Fuller and Simons, 1986). A variety of studies addressed the question of how the transferrin is reabsorbed in the kidney, regardless of its iron-loading status.

Cubilin is a 460 kDa receptor, known to bind and internalize many ligands such as the vitamin B12-intrinsic factor complex in the intestine and apo-lipoprotein in the kidney, where it is located apically on the PCT membrane. Cubilin lacks a transmembrane domain, and it builds a complex with amnionless and megalin and depends on megalin for proper localization and internalization (Christensen and Birn, 2002; Verroust and Christensen, 2002). Transferrin was found to be one of cubilin’s ligands (Kozyraki et al., 2001), suggesting that cubilin might be responsible for the reabsorption of apo- and holo-transferrin from the primary urine. In both cubilin-defective dogs and megalin-deficient mice, transferrin was detected in the urine, suggesting that, although the high affinity of transferrin is related to cubilin, megalin is probably also essential for the cubilin-mediated transferrin internalization.

To evaluate if TfR1 may play a role in transferrin reabsorption, kidney tissues where analyzed. In murine kidney sections, TfR1 was detected in the apical membrane of mouse-PCT (Zhang et al., 2007). PCT epithelium was recently shown to lack the clathrin adaptor AP-1B and therefore TfR1 is redirected to the apical rather than the basolateral membrane (Perez Bay et al., 2013). This suggests that TfR1 may also be involved in transferrin uptake and reabsorption from primary urine, but does not answer the question of the fate of the TfR1 bound transferrin. Usually this transferrin would only release its iron and recycle to the primary urine, thus further studies are needed to clarify the pathway of transferrin after its internalization. Nevertheless, reuptake of transferrin from primary urine is probably not performed exclusively by cubilin but rather in conjunction with TfR1 (Kozyraki et al., 2001).

The megalin/cubilin-complex is trafficked to the lysosome after internalization where iron is released and possibly transported to the cytosol by DMT1. In kidney-epithelium, DMT1 is mostly found intracellularly and only in distal tubules was it found on the apical membrane as well (Wareing et al., 2003). Intracellular DMT1 is likely localized to the endo/lysosomal membrane where it can mediate iron import to the cytosol after transferrin internalization by cubilin or TfR1. Research on ion transport modulation, in renal epithelial cells specifically (Welling and Weisz, 2010), can shed additional light on iron transport.

Following entry to the cytosol, iron needs to be exported to the blood through the basolateral membrane, where ferroportin is located, and may export iron to the renal interstitium. However, a recent study in mice localized ferroportin apically and suggested a role for ferroportin in iron absorption (Wolff et al., 2011; Zarjou et al., 2013).

In addition, ferritin may also play an important role in the kidney. Deletion of the H-ferritin subunit in renal PCT in mice with acute kidney injury worsened their condition (Zarjou et al., 2013). Moreover, an uneven ferritin distribution was detected in renal proximal tubule cells where ferritin was co-localized with villin and was enriched near the apical pole of the PCT-cells, a region enriched with lysosomes (Cohen et al., 2010).

## TESTIS

The testis is divided into two major compartments: (1) the looped seminiferous tubule (SFT), where spermatogenesis occurs, and (2) the interstitium composed of androgen secreting Leydig cells, blood vessels, macrophages, lymphocytes, lymphatic vessels, and connective tissue (Mital et al., 2011; Goldstein and Schlegel, 2013). In the SFT spermatocytes differentiate to mature sperm in close interaction with the Sertoli cells (SCs). SCs are connected to each other by tight junctions, forming the blood-testis barrier (BTB). The BTB plays an important role in protecting the developing spermatocytes from immune mediators (leukocytes and antibodies), toxins, pathogens, and nutritional fluctuations (Dym and Fawcett, 1970; Mital et al., 2011) and provides one of the mechanisms that maintain testis as an immune-privileged site (Arck et al., 2014).

Sertoli cells function as “nurse cells” to the developing spermatocytes (Sylvester and Griswold, 1994), surround the germ cells and thus are able to supply them with nutrients and regulatory factors while functioning as a scaffold on which the germ cells move unidirectionally toward the SFT lumen. The SFT is lined with peritubular myoid cells (PTM) that form an additional barrier and are responsible for the contraction of the tubule and the subsequent transport of maturing sperm cells and testicular fluid from the SFT lumen to the epididymis where they further mature (Maekawa et al., 1996; Goldstein and Schlegel, 2013).

The BTB is not completely impervious, as it allows the passage of developing spermatocytes from the basolateral to the adluminal compartment. This process is highly regulated, ensuring a continuing presence of the BTB and protection of the delicate developing spermatocytes (Smith and Braun, 2012).

Iron is needed by spermatocytes mainly for DNA synthesis and mitochondriogenesis. Recently, we suggested a novel model for an autonomous iron cycle within the SFT (Leichtmann-Bardoogo et al., 2012) that renders testes resistant to fluctuations of peripheral iron and ensures a constant supply of iron for maturing spermatocytes. The findings that ferritin levels were high in early spermatocytes near the basal membrane of the SFT, but mRNA levels, especially of the H- subunit were much lower in these cells than in the neighboring SCs and PTM, raised the possibility that ferritin may be imported to spermatogonia (http://public.wsu.edu/∼griswold/microarray). As the spermatocytes develop, their ferritin levels decreased toward the SFT lumen, suggesting intracellular iron redistribution into functional compartments such as the mitochondria of developing meiotic germ cells. During spermatid-maturation the elongating spermatids shed residual bodies containing cytosol and mitochondria that are phagocytosed by SCs near the SFT lumen, thus recycling the iron back to the SCs. DMT1 did not co-localize with TfR1 and was found in elongating spermatids and near the apical pole of SCs, signifying that DMT1 is involved in TfR1-independent iron transport in the SCs. SCs synthesize ferritin and we suggested that they traffic ferritin to the basolateral pole where ferritin is secreted and passed on to the early spermatocytes in a regulated manner (Leichtmann-Bardoogo et al., 2012). Supporting this concept is the polarized distribution of ferritin with increased ferritin concentration near the basolateral pole detectable in the MSC1 mouse Sertoli cell-line (Figure 1).

Ferroportin is mainly expressed on the PTM surrounding the SFT and possibly on the basal membrane of SCs. Although its role is unknown, one possibility is that it could link the SFT iron cycle to the iron rich interstitium by exporting excess toxic iron from the SFT.

## RETINA

The retinal pigment epithelium (RPE) is a monolayer of pigmented cells that form the blood–retina barrier (BRB) together with the neuro-retinal vasculature. These two cell layers, epithelial and endothelial, respectively, define the retinal compartment and separate it from the periphery. The RPE apical membrane and processes face the retinal photoreceptors’ outer segments, enabling close interaction and exchange between these two cell-types, and the RPE basal membrane faces the Bruch’s membrane that separates between RPE and the chorio-capillaries (Strauss, 2005).

TfR1 on the basal surface of RPE cells binds and takes up transferrin from the choroidal circulation into endosomal compartments. However, a full iron transport mechanism from the basal to the apical surface has not been elucidated, and TfR1 was also found on the apical membrane of RPE where it can bind and internalize retinal transferrin that is synthesized and secreted from RPE (Hunt et al., 1989; Yefimova et al., 2000). These findings suggest that the direction of iron flow through RPE may be adapted to retinal needs. *Trans*-cytosis of transferrin, release of elemental iron that originates from transferrin and release of ferritin-like molecules have all been implicated in RPE iron trafficking (Hunt et al., 1989; Hunt and Davis, 1992; Burdo et al., 2003). Elemental iron is exported from cells by ferroportin, which was detected in mouse retina in several cell types including RPE. Though in RPE it was localized near the basal surface, suggesting ferroportin involvement, along with ceruloplasmin and hephaestin, in iron export to the choroidal vasculature (He et al., 2007).

Furthermore, ferritin is distributed in a polarized manner in the RPE and was more concentrated near the basolateral pole (Hahn et al., 2004 and Figure 2B Joshua Dunaief, personal communication). In addition, the role of DMT1 in the retina and its localization in RPE remains unclear (He et al., 2007). Taken together, the physiology of iron transport across the RPE barrier awaits interesting research.

## CHOROID PLEXUS

Transport of molecules into the brain is strictly regulated by two major barrier systems: the blood–brain barrier (BBB) and the blood–cerebrospinal fluid barrier (BCB). The BBB is formed by the tight junctions of the endothelial cells, and separates the blood circulation from the brain interstitial fluid (ISF). The BCB is formed by the tight junctions of the choroid plexus (CP) epithelium and arachnoid membrane, and separates the blood from the cerebrospinal fluid (CSF; Brightman, 1977; Caplan et al., 1997; Cipolla, 2009).

Iron is essential for neuronal function, however, iron deficiency and excess are known to underlie the patho-etiology of several neurodegenerative disorders (Rouault, 2013). The uptake of iron at the BBB is well documented (Rothenberger et al., 1996; Richardson and Ponka, 1997; Fillebeen et al., 1999; Morgan and Moos, 2002; Moos and Rosengren Nielsen, 2006). In recent years, however, an iron uptake pathway through the CP was proposed to be of importance, (Marques et al., 2009; Rouault et al., 2009; Connor et al., 2011; Mesquita et al., 2012) mainly due to the combination of high surface area (Speake and Brown, 2004) and high blood supply (Maktabi et al., 1990) of the CP cells. The basolateral side of these polarized epithelial cells faces the blood, whereas the apical side contains microvilli that are in direct contact with the CSF (Abbott et al., 2006). Thus, iron transport across CP cells is an example of an epithelial barrier-transport.

CP seems to be the central expression site for the majority of iron metabolism key proteins within the brain (Rouault et al., 2009). TfR1 was expressed on the basolateral side of CP cells facing the capillaries (Moos, 2002). In another report, TfR1 was localized perinuclear while DMT1 and ferroportin were located apically (Wang et al., 2008). The ferroportin localization suggests a direction of iron flux into the CSF (Wu et al., 2004). The iron reductase duodenal cytochrome B (dcytb) was found mainly apically and ferritin distribution was strikingly different from dcytb, but could not be confirmed to show basolateral accumulation (Rouault et al., 2009). Additional studies must be performed to clarify the role of the CP in iron metabolism.

## CONCLUSION

Transferrin receptor 1 and divalent metal transporter 1 are so far the most studied iron importers and ferroportin is the main confirmed iron exporter. Information on their localization and trafficking in epithelial barriers accumulates and is suggestive for directions of iron transport across these barriers.

Still little is known about the intracellular pathway of iron across these polarized cells. In our view, it is intriguing that ferritin is not evenly distributed throughout the cytosol, appears often punctate and accumulates near specific poles of these barrier-cells (Figure 2A). It is possible that polarized ferritin resides in the endo-lysosomal system, and that the punctate accumulation of ferritin is within these subcellular compartments. In the two epithelial monolayers that separate the outside from the inside of the body, namely intestinal and renal epithelium, ferritin distribution is polarized in such a way, that it is closer to the apical side and the microvilli. Conversely, in epithelial monolayers that separate a compartment from the periphery, such as Sertoli cells and RPE, ferritin is close to the basolateral membrane (LaVaute et al., 2001; Hahn et al., 2004; Rouault et al., 2009; Cohen et al., 2010; Vanoaica et al., 2010; Leichtmann-Bardoogo et al., 2012; and demonstrated in Figures 1 and 2). In Sertoli cells, polarized ferritin may be secreted and may function as an iron exporter (Leichtmann-Bardoogo et al., 2012), which could be one of the roles of polarized ferritin. Nevertheless, the function of ferritin polarization still needs to be elucidated.

## Conflict of Interest Statement

The authors declare that the research was conducted in the absence of any commercial or financial relationships that could be construed as a potential conflict of interest.
