# Corrigendum: Ferritin polarization and iron transport across monolayer epithelial barriers in mammals

**DOI:** 10.3389/fphar.2015.00163

**Published:** 2015-08-06

**Authors:** Esther G. Meyron-Holtz, Lyora A. Cohen, Lulu Fahoum, Yael Haimovich, Lena Lifshitz, Inbar Magid-Gold, Tanja Stuemler, Marianna Truman-Rosentsvit

**Affiliations:** Laboratory for Molecular Nutrition, Faculty of Biotechnology and Food Engineering, Technion, Israel Institute of TechnologyHaifa, Israel

**Keywords:** iron transport, iron metabolism, epithelial barriers, tight junctions, ferritin polarization

On the original article the acknowledgment section was missing a funding source. The correct and updated acknowledgments are below.

## Conflict of interest statement

The authors declare that the research was conducted in the absence of any commercial or financial relationships that could be construed as a potential conflict of interest.

